# The Quality of Life in Women with Burns in Iran

**DOI:** 10.29252/wjps.8.1.33.

**Published:** 2019-01

**Authors:** Jafar Kazemzadeh, Soheila Rabiepoor, Saeedeh Alizadeh

**Affiliations:** 1 Reconstructive and Burn Surgery, Urmia University of Medical Sciences, Urmia, Iran;; 2 Nursing and Midwifery Department, Reproductive Health Research Center, Urmia University of Medical Sciences, Urmia, Iran;; 3 Nursing and Midwifery Department, Urmia University of Medical Sciences, Urmia, Iran

**Keywords:** Burn, Quality of life, Women, Iran

## Abstract

**BACKGROUND:**

Severe and extensive burns and the consequent burn scars have a profound impact on various aspects of the patients’, especially women’s quality of life (QOL). Although burn is considered as a common phenomenon in Iran, few studies are conducted to investigate the QOL in women with burn scars. Thus, the present study aimed at investigating the quality of life of married women with severe burns.

**METHODS:**

The present study was a descriptive-analytic study conducted on 101 patients with severe burns who referred in 2016 to Imam Khomeini Hospital, Tehran, Iran. The Burn Specific Health Scale (BSHS) and a demographic questionnaire were used to collect and analyze the data.

**RESULTS:**

Burns had a significant negative impact on the life quality of women with burns. The average score of quality of life in women was obtained as 51.47±10.44. The most difficult in the quality of life of the patients were in skin sensitivity to heat and treatment regimens and the least difficulty was in simple abilities and hand function.

**CONCLUSION:**

The interventions to improve the quality of life is of high importance in the patients with burns. The findings of this study can help us in designing care plans for women with burns.

## INTRODUCTION

Burn is a danger that threatens people’s lives in different ways.^1^ According to the statistics, burn injuries are among the five leading causes of death in different age groups in developing and underdeveloped countries.^2^^,^^3^ Burn is the sixth cause of death in Iran. In 2006, 48000 cases of burns that led to hospitalization were recorded.^4^ According to an epidemiological study conducted by Aghakhani *et al.*, the incidence rate of burn in West Azerbaijan, Iran was estimated as 21 cases per 100000 people. They also found that women comprised 48.36% of the burn cases.^5^

Primary care for severe burns is only a part of treatment process and most patients need several years of follow-up.^6^ Severe and extensive burns have a great negative impact on various aspects of the patients’ life.^7^^,^^8^ Burn injuries, especially the scars caused by burns, may remain for several years, or even decades, and are considered the biggest problems for the patients.^9^^,^^10^ Today, in developed countries, the quality of treatment is not measured only by the survival of the patients, rather, it is measured by the functionality and physical appearance of the burned members in the long run, and the patients’ quality of life after burn. The injuries and disabilities caused by burn can have a negative impact on the patients’ QOL.^11^

Emotional problems, Post-traumatic stress, body dysmorphic, and social anxiety disorders, skin lesions and the related complications such as muscular dystrophy, dysfunction, skin allergies and rashes are among the problems that can affect the QOL of the burn patients.^12^^-^^14^ Scars in the areas such as face and hands, that are not covered and are easily seen, can cause social anxiety disorders, especially in women.^15^ In addition, burn scars negatively affect the patients’ body image and life satisfaction.^16^ It seems that the changes in appearance that are caused by burns can negatively affect the patients, especially women’s QOL. Although burn is a common phenomenon in Iran, few studies have been conducted to investigate women’s QOL in relation to burn scars. Thus, the present study aimed at investigating the QOL of Iranian women with burns. 

## MATERIALS AND METHODS

The present descriptive-analytic study was carried out at Imam Khomeini Hospital of Urmia as the only referral center accepting burn patients in West Azerbaijan Province, northwest of Iran. A convenient sample of 101 married women who were admitted to the hospital were selected according to the criteria defined by Low *et al.*^14^ The inclusion criteria for the study were burn patients with 15% or greater total body surface area (TBSA), 6 months after discharge, age over 15 years, female, married, and with a history of admission to the hospital to treat burns. The exclusion criteria were mental retardation, communication and hearing disorders, and unwillingness to participate in the study. Research questions were (i) What are the QOL and its domains scores in women with burns? and (ii) Which demographic variables affect the QOL in women with burns?

The first instrument was a demographic questionnaire including age, residence, level of education, occupation, duration of injury, etiology, extent (TBSA burned), site of burn, and number of alive children. The second instrument was the Burn Specific Health Scale-Brief (BSHS-B). The scale was a 40-item questionnaire with the following nine domains: heat sensitivity (items 28-32), effect (items 10-16), hand function (items 4-8), treatment regimen (items 33-37), work (9 and 38-40), sexuality (21-23), interpersonal relationship (items 17-20), simple abilities (items 1-3), and body image (items 24-27). Responses were rated on a 5-point Likert scale from 0 (extremely) to 4 (non/not at all) for each of the 40 items. 

A high mean score indicated a better QOL while low scores were indicators of low QOL.^17^ The score of each domain and total score of QOL were standardized so that they could be compared. In the study by Kilda *et al.*, the obtained reliability from the Cronbach alpha coefficient for each domain showed a reliably of 0.75-0.93.^17^ The validity of the scale was confirmed for Iranian context in the study by Pishnamazy *et al.*, and its reliability was tested using test-retest and found to be 0.85.^18^ After the proposal of study was approved by the Urmia University of Medical Sciences, the data were collected from Imam Khomeini Hospital, the only referral center accepting burn patients in West Azerbaijan, Iran. The sample was comprised of 101 women who had referred to Burn Clinic of Imam Khomeini Hospital from Jun 2016 to Jan 2017.

 The patients who met the inclusion criteria, in case of their consent to take part in the study were invited to fill out the questionnaires. For the participants who were illiterate, the questionnaires were filled out by another person through an interview. The required time to fill out the questionnaire was about 20-25 minutes. The obtained data were analyzed using SPSS software (version 16, Chicago, IL, USA). Descriptive statistics as well as an ANOVA test and independent sample t test were run to analyze the obtained data. The present study was approved by and registered in the Ethics Committee of Urmia University of Medical Sciences by code no. IR.umsu.rec.1395.119. The participants were assured about the confidentiality of the data and advised that the information they share with the researcher would not be disclosed to other individuals. The researcher also assured the patients that the participation in the study was voluntary.

## RESULTS

The demographic variables in relation to burn injuries were shown in Table 1. As Table 1 illustrates, two third of the participants were in the age range of 20-40 years old (30.7% in 20-30 and 34.7% in 31-40 years old). Women who were older than 51 years had the least number of burn cases (16.8%). The duration of injury was 6-12 months in most of the cases (58.4%). Fire was reported to be the most common cause of burns (45.5%), while electricity was the least common one (3%). The majority of patients had burns within the range of 15-30% TBSA. Regarding the site of burn, in 91 of the cases, burns were in the upper arms and in 72 of the cases were in the head and neck. Only 5 of the cases reported burns in their genitals. Over two third (61.4%) of the cases reported that they earned less than average income. 

**Table 1:** The distribution of the study patients according to parameters of burns

**Items**	**Parameters of burns**		
		**N**	**%**
Age (year)	20-30	31	30.7
	31-40	35	34.7
	41-50	18	17.8
	51≤	17	16.8
Duration of injury (month)	6-12	59	58.4
	13 ≤	42	41.6
Etiology of burns	Hot fluids	41	40.6
	Flame	46	45.5
	Electricity	3	3
	Chemical materials	11	10.9
TBSA	15-30	89	88.1
	31-45	12	11.9
Site of burn	Head, face and neck	72	72.00
	Upper arms	91	91.00
	Anterior trunk	57	57.00
	Posterior trunk	7	7.00
	Lower limbs	41	41.00
	Genitalia	5	5.00
Financial situation	Not enough	62	61.4
	Fair	37	36.6
	No money problem	2	2
Number of pregnancy	0-2	50	49.5
	3≤	51	50.5
	Number of children		
	0-2	60	59.4
	3≤	41	4.6
Education	Illiterate	24	23.8
	Primary and high school	57	56.4
	College certificate and diploma	20	19.8
Occupation	Home maker	94	93.1
	Practitioner	7	6.9
Residence	Urban	66	65.3
	Rural	35	34.7

Regarding the level of education, most of the patients were in primary or high school degree (56.4%) levels. Most of the participants in the present study were home maker (93.1%) and lived in urban areas (65.3%). As Table 2 shows, most of the participants (43.6%) had problems in bathing independently and more than two third of the participants had problems in dressing independently. Based on Table 3, about half of the participants (47.5%) were bothered by the appearance of their scars and 46.5% were annoyed by their general appearance. Most of the participants wanted to forget that their appearance had changed. In addition, they reported that they were troubled by feeling of loneliness (41.6%) and depression (45.5%).

**Table 2:** Distribution of the study patients according to assessment of Burn Specific Health Scale-Brief (BSHS-B) regarding simple abilities and hand functions

**Simple abilities and hand functions** **Items**	**Extremely**		**Quite a bit**		**Moderately**		**A little bit**		**Not at all**	
	**N**	**%**	**N**	**%**	**N**	**%**	**N**	**%**	**N**	**%**
Bathing independently	6	5.9	18	17.8	44	43.6	24	23.8	9	8.9
Self-dressing	6	5.9	12	11.9	35	34.7	35	34.7	13	12.9
Getting in and out of a chair	5	5	12	11.9	17	16.8	32	31.7	35	34.7
Signing name	3	3	4	4	11	10.9	26	25.7	57	56.4
Eating with utensils	3	3	5	5	6	5.9	30	29.7	57	56.4
Tying shoelaces, bows, etc.	9	8.9	4	4	14	13.9	38	37.6	36	35.6
Picking up coins from a flat surface	5	5	4	4	12	11.9	46	45.5	34	33.7
Unlocking a door	3	3	3	3	5	5	28	27.7	62	61.4

**Table 3:** Distribution of the study patients according to assessment of Burn Specific Health Scale-Brief (BSHS-B) regarding affect and body image

**Affect and body image** **Items**	**Extremely**		**Quite a bit**		**Moderately**		**A little bit**		**Not at all**	
	**N**	**%**	**N**	**%**	**N**	**%**	**N**	**%**	**N**	**%**
Troubled by feeling of loneliness	36	35.5	42	41.6	15	14.9	7	6.9	1	1
Often feel sad or blue	29	28.7	46	45.5	19	18.8	5	5	2	2
Think that have had an emotional problem	21	20.8	44	43.6	30	29.7	3	3	3	3
Not interested in doing things with friends	18	17.8	30	29.7	37	36.6	13	12.9	3	3
Do not enjoy visiting people	16	15.8	33	32.7	31	30.7	17	16.8	4	4
Have no one to talk to about problems	15	14.9	22	21.8	34	33.7	22	21.8	8	7.9
Have feelings of being trapped or caught	20	19.8	33	32.7	33	32.7	13	12.9	2	2
Would like to forget that my appearance has changed	33	32.7	47	46.5	14	13.9	3	3	4	4
Feel that burn is unattractive to others	34	33.7	39	38.6	19	8.8	4	4	5	5
Bothers by general appearance	47	46.5	36	35.6	12	11.9	3	3	3	3
Bothers by the appearance of scars	48	47.5	43	42.6	6	5.9	2	2	2	2

Table 4 demonstrates that about two fifth of the participants believed their injury had put them further away from their family. More than 90% of the participants felt frustrated since they could not be sexually aroused as well as they used to. Besides, 95% of the participants stated that they were not interested in sex anymore and 94% reported that they did not like to hug, hold, or kiss. Table 5 revealed that about half of the participants reported being out in the sun as well as hot weather bothers them. Besides, half of the participants stated that their skin was much more sensitive than before.

**Table 4:** Distribution of the study patients according to assessment of ‘Burn Specific Health Scale-Brief (BSHS-B) as regards interpersonal relationship and sexuality

**Interpersonal relationship and sexuality** **Items**	**Extremely**		**Quite a bit**		**Moderately**		**A little bit**		**Not at all**	
	**N**	**%**	**N**	**%**	**N**	**%**	**N**	**%**	**N**	**%**
Injury has put patients further away from family	10	9.9	21	20.8	42	41.6	23	22.8	5	5
Would rather be alone than with family	9	8.9	27	26.7	34	33.7	23	22.8	8	7.9
Do not like the way family acts around me	11	10.9	25	24.8	29	28.7	31	30.7	5	5
Family would be better off without me	5	5	21	20.8	31	30.7	34	33.7	10	9.9
Feel frustrated because cannot be sexually aroused as well as I used to	24	23.8	47	46.5	19	18.8	8	7.9	3	3
Not interested in sex any more	33	32.7	41	40.6	22	21.8	4	4	1	1
No longer hug, hold or kiss	35	34.7	43	42.6	17	16.8	4	4	2	2

**Table 5:** Distribution of the study patients according to assessment of Burn Specific Health Scale-Brief (BSHS-B) as regards heat sensitivity and treatment regimen

**Heat sensitivity and treatment regimens ** **Items**	**Extremely**		**Quite a bit**		**Moderately**		**A little bit**		**Not at all**	
	**N**	**%**	**N**	**%**	**N**	**%**	**N**	**%**	**N**	**%**
Being out in the sun bothers me	51	50.5	41	40.6	5	5	1	1	3	3
Hot weather bothers me	51	50.5	41	40.6	7	6.9	0	0	2	2
Can’t get out and do things in hot weather	46	45.5	42	41.6	10	9.9	1	1	2	2
Bothers me can’t get out in the sun	45	44.6	42	41.6	12	11.9	0	0	2	2
The skin is more sensitive than before	51	50.5	39	38.6	8	7.9	2	2	1	1
Taking care of skin is a bother	29	28.7	41	40.6	21	20.8	7	6.9	3	3
There are things that have been told to do for burn that I dislike doing	31	30.7	37	36.6	22	21.8	10	9.9	1	1
Wish that did not have many things to do to take care of burn	27	26.7	41	40.6	25	24.8	6	5.9	2	2
Have a hard time doing all the things have been told to take care of burn	26	25.7	35	34.7	32	31.7	6	5.9	2	2
Taking care of burn makes it hard to do other things that are important	20	19.8	43	42.6	27	26.7	8	7.9	3	3

As it can be seen in Table 6, about half of the participants reported that burn created a lot of problems in their work, while, only 5.9% found that they did not face any problems in their work. About two third of the participants reported that burn interfered with their work. According to the findings presented in Table 7, heat sensitivity, treatment regimens, and interpersonal and affect domains had the lowest scores among the participants with mean scores of 33.5±13.58, 44±16.19, 44.1±14, respectively. On the other hand, the highest domain score was seen in simple abilities and hand function with mean score of 77.89±13.9. The overall average score for QOL in women with burns was 51.47±10.44.

**Table 6:** Distribution of the study patients according to assessment of Burn Specific Health Scale-Brief (BSHS-B) as regards work

**Work ** **Items**	**Extremely**		**Quite a bit**		**Moderately**		**A little bit**		**Not at all**	
	**N**	**%**	**N**	**%**	**N**	**%**	**N**	**%**	**N**	**%**
Burn interferes with work	18	17.8	42	41.6	31	30.7	7	6.9	3	3
Being burned has affected ability to work	21	20.8	45	44.6	26	25.7	5	5	4	4
Burn has caused problems with working	22	21.8	48	47.5	19	18.8	6	5.9	6	5.9
How much difficulty do you have working in your old job performing your old duties?	8	7.9	22	21.8	34	33.7	29	28.7	8	7.9

**Table 7:** Distribution of the study patients according to Burn Specific Health Scale-Brief (BSHS-B) total scores, mean and standard deviations

**Total score**				
**Variables**	**Minimum**	**Maximum**	**Mean**	**SD**
Simple abilities and hand function	37.5	100	77.89	13.9
Affect and body image	20	100	44.10	14
Interpersonal relationship and sexuality	22.86	100	51.96	15.4
Heat sensitivity	20	100	33.50	13.58
Treatment regimens	20	92	44	16.19
Work	20	100	49.8	15.68
Overall average score for BSHS-B	29.50	96.5	51.47	10.44

Table 8 exhibits that there was a significant relationship between interpersonal relationship-sexuality and age (*p*=0.004). However, no significant relationship was observed between age and other domains of QOL as well as overall score of QOL in women with burns. Table 9 demonstrates that there was a statistical significant relationship between residence and heat sensitivity (*p*=0.003) as well as treatment regimens (*p*=0.04), in that, women from rural areas had lower scores as compared with the women who lived in cities.

**Table 8:** Correlation between Burn Specific Health Scale-Brief (BSHS-B) and socio demographic data of burn patients related to age

**BSHS-B domains**	**Age**										**F**	**P value**
	**20-30**		**31-40**		**41-50**		**51≤**		**Total**			
	**Mean **	**SD**	**Mean **	**SD**	**Mean **	**SD**	**Mean **	**SD**	**Mean **	**SD**		
Simple abilities and hand function	31.90	5.16	30.22	6.48	30.88	4.52	32.00	5.32	31.15	5.56	0.64	0.58
Affect and body image	22.96	8.29	26.57	7.91	21.72	6.25	24.52	6.71	24.25	7.70	2.06	3.16
Interpersonal relationship and sexuality	17.12	5.45	20.65	5.20	15.66	5.09	17.70	4.22	18.18	5.39	4.66	0.004*
Heat sensitivity	8.9	4.64	8.08	2.79	8.38	2.47	8.00	2.78	8.37	3.39	0.37	0.77
Treatment regimens	10.80	3.73	11.57	4.79	10.38	3.18	10.82	3.92	11	4.04	0.39	0.75
work	9.67	3.3	10.08	3.64	10.11	2.34	10.05	2.58	9.96	3.13	0.11	0.94
Overall average score for BSHS-B	101.38	23.67	107.2	21.18	97.16	16.45	103.11	18.72	102.94	20.88	1.002	0.39

F: ANOVA test; SD: Standard deviation;

* Statistically significant (p<0.05).

**Table 9:** Correlation between Burn Specific Health Scale-Brief (BSHS-B) and socio-demographic data of burn patients related to residence

**BSHS-B domains**	**Residence**						**P value**
	**Rural**		**Urban**		**Total**		
	**Mean**	**SD**	**Mean**	**SD**	**Mean**	**SD**	
Simple abilities and hand function	30.11	5.54	31.71	5.53	31.15	5.56	0.17
Affect and body image	23.77	8.79	24.51	7.11	24.25	7.7	0.64
Interpersonal relationship and sexuality	18.08	6.19	18.24	4.96	18.18	5.39	0.89
Heat sensitivity	7	2	9.1	3.75	8.37	3.39	0.003*
Treatment regimens	9.88	3.99	11.59	3.98	11	4.04	0.04*
Work	9.37	3.07	10.27	3.14	9.96	3.13	0.17
Overall average score for BSHS-B	98.22	21.83	105.43	20.07	102.94	20.88	0.09

t: Independent sample t test; SD: Standard deviation;

* Statistically significant (*p*<0.05).

Table 10 illustrates that there was no statistically significant correlation between level of education and different domains of QOL in women with burns. Table 11 indicates that there was a significant relationship between TBSA burnt and overall average score of QOL (*p*=0.007). The results also denotes to a significant relationship in the following domains: simple abilities and hand function (*p*=0.02), affect and body image (*p*=0.03), and interpersonal relationship and sexuality (*p*=0.005), the higher the surface area of burn, the lower the QOL score in these domains. Table 12 reveals that there was not any significant relationship between overall QOL score as well as average scores in different domains and duration of injury. Table 13 presents a significant relationship between economic status and overall average score of QOL in the participants (*p*=0.02). However, no significant relationship was observed between different domains of QOL and economic status.

**Table 10:** Correlation between Burn Specific Health Scale-Brief (BSHS-B) and socio-demographic data of burn patients related to education

**BSHS-B domains**	**Educational level**								**F**	**P value**
	**Illiterate**		**Primary, High school**		**College certificate, diploma**		**Total**			
	**Mean**	**SD**	**Mean**	**SD**	**Mean**	**SD**	**Mean**	**SD**		
Simple abilities and hand function	30.95	5.98	30.98	5.48	31.9	5.47	31.15	5.56	0.21	0.80
Affect and body image	26.41	10.19	23.36	6.46	24.2	7.42	24.25	7.7	1.33	0.26
Interpersonal relationship and sexuality	18.87	6.05	18.10	4.99	17.6	5.84	18.18	5.39	0.31	0.73
Heat sensitivity	9.04	4.99	8.08	2.76	8.4	2.68	8.37	3.39	0.66	0.51
Treatment regimens	11.87	4.74	10.61	4.18	11.05	2.43	11	4.04	0.81	0.44
Work	10.7	1.09	9.61	2.9	10.05	2.35	9.96	3.13	1.03	0.35
Overall average score for BSHS-B	107.87	27.65	100.77	18.47	103.20	17.84	102.94	20.88	0.97	0.37

F: ANOVA test; SD: Standard deviation.

**Table 11:** Correlation between Burn Specific Health Scale-Brief (BSHS-B) and parameters of burn patients related to total surface area burn

**BSHS-B domains**	**Burn surface area**						**P value**
	**15-30**		**31-45**		**Total**		
	**Mean **	**SD**	**Mean **	**SD**	**Mean **	**SD**	
Simple abilities and hand function	31.61	5.56	27.75	4.33	31.15	5.56	0.02*
Affect and body image	24.86	7.74	19.75	5.92	24.25	7.7	0.03*
Interpersonal relationship and sexuality	18.71	5.33	14.25	4.2	18.18	5.39	0.006*
Heat sensitivity	8.52	3.46	7.25	2.73	8.37	3.39	0.22
Treatment regimens	11.17	4.10	9.66	3.49	11	4.04	0.22
Work	10.07	3.23	9.08	2.15	9.96	3.13	0.30
Overall average score for BSHS-B	104.98	20.78	87.75	14.93	102.94	20.88	0.007*

t: Independent sample t test; SD: Standard deviation;

* Statistically significant (*p*<0.05).

**Table 12:** Correlation between Burn Specific Health Scale-Brief (BSHS-B) and parameters of burn patients related to Duration of injury (month)

**BSHS-B domains**	**Duration of injury (month)**						**T**	**P value**
	**6-12**		**13≤**		**Total**			
	**Mean**	**SD**	**Mean**	**SD**	**Mean**	**SD**		
Simple abilities and hand function	31.20	5.83	31.09	5.22	31.15	5.56	-1.39	0.16
Affect and body image	24.77	8.08	23.52	7.16	24.25	7.7	-0.39	0.69
Interpersonal relationship and sexuality	18.20	5.6	18.16	5.15	18.18	5.39	-0.45	0.65
Heat sensitivity	8.45	3.25	8.26	3.62	8.37	3.39	-0.48	0.62
Treatment regimens	11	3.5	11	4.7	11	4.04	-0.69	0.48
Work	10.08	3.04	9.78	3.28	9.96	3.13	-1.46	0.14
Overall average score for BSHS-B	103.72	21.45	102.83	20.24	102.94	20.88	-1.06	0.28

t: Independent sample t test; SD: Standard deviation.

**Table 13:** Correlation between Burn Specific Health Scale-Brief (BSHS-B) and parameters of burn patients related to financial situation

**BSHS-B domains**	**Financial situation**								**F**	**P value**
	**Not enough**		**Fair**		**No money problem**		**Total**			
	**Mean **	**SD**	**Mean **	**SD**	**Mean **	**SD**	**Mean **	**SD**		
Simple abilities and hand function	30.17	5.6	32.78	5.16	31.5	7.77	31.15	5.56	2.63	0.07
Affect and body image	22.95	7.25	26.13	8.15	30	5.56	24.25	7.7	2.68	0.07
Interpersonal relationship and sexuality	17.74	5.56	18.89	5.21	19	2.82	18.18	5.39	0.54	0.58
Heat sensitivity	7.8	2.81	9.29	4.08	9	4.24	8.37	3.39	2.32	0.1
Treatment regimens	10.33	3.74	12.02	4.45	12.5	0.7	11	4.04	2.2	0.11
Work	9.51	2.52	10.62	3.9	11.5	3.53	9.96	3.13	1.7	0.18
Overall average score for BSHS-B	98.53	19.11	109.75	22.41	113.5	7.77	102.94	20.88	3.81	0.02*

F: ANOVA test; SD: Standard deviation;

* Statistically significant (*p*<0.05).

## DISCUSSION

As the results of the present study indicated, the majority of the patients were within the age range of 20-41 years, which is in line with the findings of others.^19^^,^^20^ The present study is also in agreement with the findings of another study, that the majority of the patients were affected by flame, and followed by scalds.^21^ It was also found that the lowest domain scores were for heat sensitivity, treatment regimens, affect, and interpersonal relationships. According to the findings of Kildal *et al.*, heat sensitivity, work, and body image were the most influential domains on the patients QOL.^22^

In a study conducted by Elsherbiny *et al.*, however, the highest average scores were recorded for interpersonal relationships and sexuality as well as simple abilities and hand function. The findings of the present study are in agreement with these findings, that is, the highest average domain scores were for simple abilities and hand function as well as interpersonal relationships and sexuality. The results of the study conducted by Pishnamazy *et al.* indicated that the average score of QOL in physical domains were the highest, while the average scores in psychological domains were the lowest.^18^ The findings of the present study are in line with the findings of the study by Pishnamazy *et al.* in the physical domain, but not in psychological domain. The changes in the patients’ appearance and functionality can have physical, psychological, economic, and social consequences for the patients.

The findings of the present study revealed no significant relationship between overall average score of QOL and the patients’ age, which is in line with the findings of Pishnamazy *et al.*^18^ and Elsherbiny *et al.*^23^ This may indicate the significant impact of burns on the QOL of women in all age groups. According to the results of the present study, there was not any significant relationship between the patients’ level of education and QOL. These results contradict Elsherbiny *et al.*^23^ and Pishnamazy *et al.*^18^ who concluded that the QOL in patients with higher level of education was higher. This can be justified considering the fact that more than two third of the patients in the present study had a low level of education and there were just a few number of patients with a university degree.

According to the findings of the present study, there was a significant relationship between the circumstances of burns and the four domains of simple abilities and hand function, affect and body image, treatment regimens, and work. This is supported by Elsherbiny *et al.* who found that a significant relationship existed between circumstances of burns and simple abilities and hand function, work, as well as overall average score of QOL.^23^ The findings of the present study indicated that there was a significant relationship between overall average score of QOL and TBSA. Patients with greater burn surface area had a lower QOL. The findings also indicated that total burn surface area was significantly associated with difference in domain scores of simple abilities and hand function, affection and body image, and interpersonal relationships and sexuality. This is in line with the findings of Druery *et al.* who found that there was a significant relationship between burn TBSA and QOL.^24^ The findings of the study by Elsherbiny and colleagues also suggested a decrease in QOL in the patients with greater burn TBSA.^23^

No significant relationship was observed between duration of injury and overall average score of QOL. This finding was in agreement with Elsherbiny *et al.*^7^ It seems that the patients never got accustomed to the changes in their appearance and the complications resulted from burns. Concerning the correlation between economic status and QOL, the results revealed that lower economic status in the patients results in lower QOL. Dyster-Aas *et al.* found that the patients who did not have a good job with a decent salary had a lower score in all QOL domains as compared with the patients who had a good job with a decent salary.^25^ These findings are in line with the findings of the present study. It seems that having a good economic condition is an important factor in QOL of the patients, since the patients with good economic condition can better afford the cost of the treatments and thus receive better care.

The results of the study revealed that women with burns had a rather lower QOL, thus, interventions to improve the QOL in these patients are of utmost importance. It is recommended that interventional researches to be designed and conducted, taking the specific needs of women into account, in order to improve the quality of women’s lives. Considering the high level of sexual problems in these patients and the patients’ low average score in interpersonal relationship and sexuality domains, it is recommended that further studies to be conducted investigating the sexual performance and satisfaction of the married women.
